# A comprehensive evaluation of multicategory classification methods for microbiomic data

**DOI:** 10.1186/2049-2618-1-11

**Published:** 2013-04-05

**Authors:** Alexander Statnikov, Mikael Henaff, Varun Narendra, Kranti Konganti, Zhiguo Li, Liying Yang, Zhiheng Pei, Martin J Blaser, Constantin F Aliferis, Alexander V Alekseyenko

**Affiliations:** 1 Center for Health Informatics and Bioinformatics, New York University Langone Medical Center, 227 East 30th Street, New York, NY, USA; 2 Department of Medicine, New York University School of Medicine, 550 First Ave, New York, NY, USA; 3 Department of Pathology, New York University School of Medicine, 550 First Ave, New York, NY, USA; 4 Department of Microbiology, New York University School of Medicine, 550 First Ave, New York, NY, USA; 5 Department of Pathology and Laboratory Medicine, Department of Veterans Affairs New York Harbor Healthcare System, 423 East 23rd Street, New York, NY, USA; 6 Medical Service, Department of Veterans Affairs New York Harbor Healthcare System, 423 East 23rd Street, New York, NY, USA; 7 Whole Systems Genomics Initiative, Texas A&M University, Kleberg Center, Mail Stop 2470, College Station, TX, USA; 8 Department of Biostatistics, Vanderbilt University, 1161 21st Ave South, Nashville, TN, USA

**Keywords:** Microbiomic data, Machine learning, Classification, Feature selection

## Abstract

**Background:**

Recent advances in next-generation DNA sequencing enable rapid high-throughput quantitation of microbial community composition in human samples, opening up a new field of microbiomics. One of the promises of this field is linking abundances of microbial taxa to phenotypic and physiological states, which can inform development of new diagnostic, personalized medicine, and forensic modalities. Prior research has demonstrated the feasibility of applying machine learning methods to perform body site and subject classification with microbiomic data. However, it is currently unknown which classifiers perform best among the many available alternatives for classification with microbiomic data.

**Results:**

In this work, we performed a systematic comparison of 18 major classification methods, 5 feature selection methods, and 2 accuracy metrics using 8 datasets spanning 1,802 human samples and various classification tasks: body site and subject classification and diagnosis.

**Conclusions:**

We found that random forests, support vector machines, kernel ridge regression, and Bayesian logistic regression with Laplace priors are the most effective machine learning techniques for performing accurate classification from these microbiomic data.

## Background

Advances in low-cost, high-throughput DNA sequencing technologies have enabled the studies of the composition of microbial communities at unprecedented throughput levels. Such studies are particularly interesting for biomedicine because for every human cell in the body there are about ten microbial cells in the gut alone [1]. These microbial symbionts contribute a meta-genome to human biology and interact with the human host to perform a multitude of functions ranging from basic metabolism to immune system development. Therefore, it is conceivable that the study of microbial compositions will yield important clues in understanding, diagnosing, and treating diseases by inferring the contribution of each constituent of microbiota to various disease and physiological states.

A typical microbiomic study relies on a marker gene (or a group of markers) that can be used for the identification and quantitation of the microbes present in a given specimen. A good marker gene needs to have three essential properties: (i) it must be present in all of the microbes that we try to identify, (ii) its sequences should be conserved in members of the same species, and (iii) the interspecies difference in the gene sequence should be sufficiently significant to allow for taxonomical discrimination. The 16S rRNA gene is commonly used in microbiomic studies as a marker gene to generate human microbiota surveys. For every sample in a dataset, a human microbiota survey contains hundreds of thousands or millions of DNA sequences from the underlying microbial community. Abundances of operational taxonomic units (OTUs), extracted from the high-throughput sequencing data using upstream bioinformatic processing pipelines, can serve as input features for machine learning algorithms.

A necessary prerequisite for the creation of successful microbiomics-based models is a solid understanding of the relative strengths and weaknesses of available machine learning and related statistical methods. Prior work by Knights *et al*. took an excellent first step in this direction and established the feasibility of creating accurate models for classification of body sites and subject identification [2]. The present work extends prior research by: (i) addressing diagnostic/personalized medicine applications in addition to classification of body sites and subjects, (ii) evaluating a large number of machine learning classification and feature/OTU selection methods, (iii) using more powerful multicategory classifiers based on a one-versus-rest scheme [3,4], (iv) measuring classification accuracy by a metric that is insensitive to prior distribution of classes, and (v) performing formal statistical comparison among classifiers. The present study thus allows determination of the classifiers that perform best for microbiomic data among the many available alternatives. It also allows identification of the best performing combinations of classification and feature/OTU selection algorithms across most microbiomic datasets.

We undertook a rigorous comparison of 18 major machine learning methods for multicategory classification, 5 feature/OTU selection methods, and 2 accuracy metrics using 8 datasets spanning 1,802 human samples and various classification tasks: body site and subject classification and diagnosis. We focused here on supervised classification methods because unsupervised methods (such as clustering and principal component analysis), which are designed to reveal structure of the data, provide visual summaries, and help quality control, are not optimal (and depending on application may also be completely inadvisable) for predictively linking the data to specific response variables/phenotypes [5,6]. We found that random forests, support vector machines, kernel ridge regression, and Bayesian logistic regression with Laplace priors are the most effective machine learning techniques in performing accurate classification from microbiomic data.

## Methods

### Datasets and data preparatory steps

In this work, we used eight microbiomic datasets (Table 1). All datasets were 16S rRNA gene surveys obtained with 454 pyrosequencing. The datasets CBH, CS, CSS, FS, FSH were obtained from the study of Knights *et al*. [2] and originate from the works of Costello *et al*. [7] [SRA:ERP000071] and Fierer *et al*. [8] [SRA: SRA0102034.1]. The dataset BP was obtained from the laboratory of Martin J. Blaser (Alekseyenko AV, Perez-Perez GI, D’Souza A, Strober B, Gao Z, Methe B, Blaser MJ: Population differentiation of the cutaneous microbiota in psoriasis, forthcoming), [9] and the datasets PBS and PDX were obtained from the laboratory of Zhiheng Pei [10] at New York University (NYU) Langone Medical Center.

**Table 1:** Characteristics of microbiomic datasets used in this study

**Dataset**	**Number of samples**	**Number of features (OTUs)**	**Number of classes**	**Classification task and samples per class**	**Max. prior probability of a class (%)**
*Costello Body Habitat (CBH)*	552	6,979	6	Classify body habitats: Skin (357), Oral Cavity (46), External Auditory Canal (44), Hair (14), Nostril (46), Feces (45)	64.7
*Costello Subject (CS)*	140	2,543	7	Classify 7 subjects by microbiota (20/20/20/20/20/20/20)	14.3
*Costello Skin Sites (CSS)*	357	4,793	12	Classify skin sites: external nose (14), forehead (32), glans penis (8), labia minora (6), axilla (28), pinna (27), palm (64), palmar index finger (28), plantar foot (64), popliteal fossa (46), volar forearm (28), umbilicus (12)	17.9
*Fierer Subject (FS)*	104	1,217	3	Classify 3 subjects by microbiota (40/33/31)	38.5
*Fierer Subject x Hand (FSH)*	98	1,217	6	Classify by subject and left/right hand (20/18/17/14/16/13)	20.4
*Blaser Psoriasis (BP)*	151	13,503	3	Classify as Control (49), Psoriasis Normal (51), Psoriasis Lesion (51)	33.8
*Pei Diagnosis (PDX)*	200	74,018	4	Classify as Normal (28), Reflux Esophagitis (36), Barrett's Esophagus (84), Esophageal Adenocarcinoma (52)	42.0
*Pei Body Site (PBS)*	200	74,018	4	Classify body site: Oral Cavity (51), Esophagus (51), Stomach (48), Stool (50)	25.5

A major data preparatory step in the analysis of human microbiota gene surveys is the extraction of operational taxonomic units (OTUs) that serve as input features for machine learning algorithms. An OTU is a cluster of sequences of non-human origin that is constructed based on nucleotide similarity between the sequences. The necessity to use sequence similarity-based OTUs is motivated by two major considerations: (i) good reference databases may not be available for fine-grained taxonomic classification of sequences, and (ii) sequencing errors introduced by the technologies are effectively controlled when sequences are aggregated into similarity-based clusters.

The OTUs were constructed using UCLUST software version 1.2.22q (http://www.drive5.com/usearch/, [11]) at a sequence similarity threshold of 97%, as recommended in the study [2]. UCLUST was applied after processing the raw DNA sequencing data with the Quantitative Insights Into Microbial Ecology (QIIME) pipeline version 1.3.0 (http://qiime.org/), which is specifically designed for high-throughput 16S rRNA sequencing studies [12]. All parameter values used for processing are provided in Table 2.

**Table 2:** **Values of parameters of the preprocessing methods**[2,11,12]

**Parameter**	**Value**	**Description**
*otu picking method*	uclust	uclust, creates ‘seeds’ of sequences which generate clusters based on percent identity.
*clustering algorithm*	furthest	Clustering algorithm for mothur otu picking method. Valid choices are: furthest, nearest, average.
*max cdhit memory*	400	Maximum available memory to cd-hit-est (via the program’s -M option) for cdhit OTU picking method (units of Mbyte)
*refseqs fp*	None	Path to reference sequences to search against when using -m blast, -m uclust_ref, or -m usearch_ref
*blast db*	None	Pre-existing database to blast against when using -m blast
*similarity*	0.97	Sequence similarity threshold (for cdhit, uclust, uclust_ref, or usearch)
*max e value*	1.00E-10	Max E-value when clustering with BLAST
*prefix prefilter length*	None	Prefilter data so seqs with identical first prefix_prefilter_length are automatically grouped into a single OTU
*trie prefilter*	FALSE	Prefilter data so seqs which are identical prefixes of a longer seq are automatically grouped into a single OTU
*prefix length*	50	Prefix length when using the prefix_suffix otu picker
*suffix length*	50	Suffix length when using the prefix_suffix otu picker
*optimal uclust*	FALSE	Pass the -optimal flag to uclust for uclust otu picking.
*exact uclust*	FALSE	Pass the -exact flag to uclust for uclust otu picking.
*user sort*	FALSE	Do not assume input is sorted by length
*suppress presort by abundance uclust*	FALSE	Suppress presorting of sequences by abundance when picking OTUs with uclust or uclust_ref
*suppress new clusters*	FALSE	Suppress creation of new clusters using seqs that don’t match reference when using -m uclust_ref or -m usearch_ref
*suppress uclust stable sort*	FALSE	Do not pass -stable-sort to uclust
*max accepts*	20	Max_accepts value to uclust and uclust_ref
*max rejects*	500	Max_rejects value to uclust and uclust_ref
*word length*	12	W value to usearch, uclust, and uclust_ref. Set to 64 for usearch.
*stepwords*	20	Stepwords value to uclust and uclust_ref
*suppress uclust prefilter exact match*	FALSE	Do not collapse exact matches before calling uclust

In summary, we started with raw DNA sequencing data, removed human DNA sequences, defined OTUs over microbial sequences, and quantified relative abundance of all sequences that belong to each OTU. These relative abundances, further rescaled to the range [0, 1], served as input features for machine learning algorithms. The number of OTUs in each dataset is provided in Table 1. We emphasize that the OTUs were constructed without knowledge of classification labels and thus do not bias performance of machine learning techniques.

The OTU tables and sample labels for all datasets used in the study are provided in Additional files 1, 2, 3, 4, 5, 6, 7 and 8.

### Machine learning algorithms for classification

We used 18 machine learning multicategory classification algorithms from the following seven algorithmic families: support vector machines, kernel ridge regression, regularized logistic regression, Bayesian logistic regression, random forests, k-nearest neighbors, and probabilistic neural networks. These machine learning methods were chosen because of their extensive and successful applications to many datasets from other genomic domains. Since all the classification tasks were multicategory (that is, with three or more classes) and most of the employed classifiers (except for random forests, k-nearest neighbors, and probabilistic neural networks) are designed for binary classification problems (that is, with two classes), we adopted a one-versus-rest approach for the latter methods. Specifically, we trained separate binary classifiers for each class against the rest and then classified new samples by taking a vote of the binary classifiers and choosing the class with the ‘strongest’ vote. The one-versus-rest approach for classification is known to be among the best performing methods for multicategory classification for other types of data, including microarray gene expression [3,4]. Random forests, k-nearest neighbors, and probabilistic neural networks methods can solve multicategory problems natively and were applied directly.

*Support vector machines* (SVMs) are a class of machine learning algorithms that perform classification by separating the different classes in the data using a maximal margin hyperplane [13]. To learn non-linear decision boundaries, SVMs implicitly map the data to a higher dimensional space by means of a kernel function, where a separating hyperplane is then sought. The superior empirical performance of SVMs in many types of high-throughput biomedical data can be explained by several theoretical reasons: for example, SVMs are robust to high variable-to-sample ratios and large numbers of features, they can efficiently learn complex classification functions, and they employ powerful regularization principles to avoid overfitting [3,14]. Extensive literature on applications in text categorization, image recognition and other fields also show the excellent empirical performance of this classifier in many other domains. SVMs were used with linear kernel, polynomial kernel, and a radial basis function (RBF, also known as Gaussian) kernel.

*Kernel ridge regression* (KRR) adds the kernel trick to ridge regression. Ridge regression is linear regression with regularization by an L_2_ penalty. Kernel ridge regression and SVMs are similar in dealing with non-linearity (by using the kernel trick) and model regularization (by using an L_2_ penalty, also called the ridge). The difference lies in the loss function: the SVMs use a hinge loss function, while ridge regression uses squared loss [15].

*Regularized Logistic Regression* adds regularization by an L_1_ or L_2_ penalty to the logistic regression (abbreviated as L1-LR and L2-LR, respectively) [16,17]. Logistic regression is a learning method from the class of general linear models that learns a set of weights that can be used to predict the probability that a sample belongs to a given class [18]. The weights are learned by minimizing a log-likelihood loss function. The model is regularized by imposing an L_1_ or L_2_ penalty on the weight vector. An L_2_ penalty favors solutions with relatively small coefficients, but does not discard any features. An L_1_ penalty shrinks the weights more uniformly and can set weights to zero, effectively performing embedded feature selection.

*Bayesian logistic regression* (BLR) is another method from the class of general linear models that finds the maximum *a posteriori* estimate of the weight vector under either Gaussian or Laplace prior distributions, using a coordinate descent algorithm [19,20]. Gaussian priors tend to favor dense weight vectors, whereas Laplace priors lead to sparser solutions; in this way they perform a similar purpose as imposing an L_1_ penalty on the coefficients.

*Random forests* (RF) is a classification algorithm that uses an ensemble of unpruned decision trees, each built on a bootstrap sample of the training data using a randomly selected subset of features [21]. The random forest algorithm possesses a number of appealing properties, making it well-suited for classification of microbiomic data: (i) it is applicable when there are more predictors than observations; (ii) it performs embedded feature selection and it is relatively insensitive to the large number of irrelevant features; (iii) it incorporates interactions between predictors: (iv) it is based on the theory of ensemble learning that allows the algorithm to learn accurately both simple and complex classification functions; (v) it is applicable for both binary and multicategory classification tasks; and (vi) according to its inventors, it does not require much fine tuning of parameters and the default parameterization often leads to excellent classification accuracy [21].

*K-nearest neighbors (KNN)* algorithm treats all objects as points in *m*-dimensional space (where *m* is the number of features) and given an unseen object, the algorithm classifies it by a vote of K nearest training objects as determined by some distance metric, typically Euclidian distance [15,22].

*Probabilistic Neural Networks (PNN)* belong to the family of Radial Basis Function (RBF) neural networks [22], and are composed of an input layer, a hidden layer consisting of a pattern layer and a competitive layer, and an output layer (see [23,24]). The pattern layer contains one unit for each object in the training dataset. Given an unseen training object, each unit in the pattern layer computes a distance from this object to objects in the training set and applies a Gaussian density activation function. The competitive layer contains one unit for each classification category, and these units receive input only from pattern units that are associated with the classification category to which the training object belongs. Each unit in the competitive layer sums over the outputs of the pattern layer and computes a probability of the object belonging to a specific classification category. Finally, the output unit corresponding to the maximum of these probabilities outputs ‘1’, while those remaining output ‘0’.

Table 3 describes software implementations for each classifier.

**Table 3:** Parameters and software implementations of the classification algorithms

**Method**	**Parameter**	**Value**	**Software implementation**
*SVM, Linear default*	*C* (penalty parameter)	1	libsvm [25,26] (http://www.csie.ntu.edu.tw/~cjlin/libsvm/)
*SVM, Linear optimized*	*C* (penalty parameter)	optimized over (0.01, 0.1, 1, 10, 100)	
*SVM, Polynomial*	*C* (penalty parameter)	optimized over (0.01, 0.1, 1, 10, 100)	
	*q* (polynomial degree)	optimized over (1, 2, 3)	
*SVM, RBF*	*C* (penalty parameter)	optimized over (0.01, 0.1, 1, 10, 100)	
	γ (determines RBF width)	optimized over (0.01, 0.1, 1, 10, 100)/number of variables	
*KRR, Polynomial*	*λ* (ridge)	optimized over (10^-10^, 10^-9^, …, 1)	clop [15,27,28] (clopinet.com/CLOP/)
	*q* (polynomial degree)	optimized over (1, 2, 3)	
*KRR, RBF*	*λ* (ridge)	optimized over (10^-10^, 10^-9^, …, 1)	
	γ (determines RBF width)	optimized over (0.01, 0.1, 1, 10, 100)/number of variables	
*KNN, default K = 1*	*K* (number of neighbors)	1	Matlab Statistics Toolbox (http://www.mathworks.com)
*KNN, default K = 5*	*K* (number of neighbors)	5	
*KNN, optimized*	*K* (number of neighbors)	optimized over (1, …, 50)	
*PNN*	*σ* (spread)	optimized over (0.01, 0.02, …, 1)	Matlab Neural Network Toolbox (http://www.mathworks.com)
*L2-LR, default*	*C* (penalty parameter)	1	liblinear [16,17] (http://www.csie.ntu.edu.tw/~cjlin/liblinear/)
*L2-LR, optimized*	*C* (penalty parameter)	optimized over (0.01, 0.1, 1, 10, 100)	
*L1-LR, default*	*C* (penalty parameter)	1	
*L1-LR, optimized*	*C* (penalty parameter)	optimized over (0.01, 0.1, 1, 10, 100)	
*BLR, Gaussian priors*	*v* (variance)	automatically determined in the software by cross-validation	bbr (http://www.bayesianregression.org)
*BLR, Laplace priors*	*v* (variance)	automatically determined in the software by cross-validation	
*RF, default*	*ntree* (number of trees)	500	R package randomForest (cran.r-project.org/)
	*mtry* (number of variables sampled at each split)	$\sqrt{\mathrm{number}\;\mathrm{of}\;\mathrm{variables}}$	
*RF, optimized*	*ntree* (number of trees)	optimized over (500, 1000, 2000)	
	*mtry* (number of variables sampled at each split)	$\mathrm{optimized}\;\mathrm{over}\;\left(0.5,1,2\right)\;\times \;\sqrt{\mathrm{number}\;\mathrm{of}\;\mathrm{variables}}$	

### Parameters of machine learning classification algorithms

Parameters for the classification algorithms were selected by the nested cross-validation procedure that is mentioned in the following subsection. We also included classifiers with default parameters for comparison purposes. For SVMs and KRR, we used the following polynomial kernel:

$$K\left(x,y\right)={\left(x^{T}y+1\right)}^{q}$$

and RBF kernel:

$$K\left(x,y\right)=\exp\left(-\gamma {\left\|x-y\right\|}^{2}\right),$$

where *x* and *y* are samples with sequence abundances and *q* and γ are kernel parameters. Table 3 describes the parameter values for each classifier.

### Model/parameter selection and accuracy estimation strategy

For model/parameter selection and accuracy estimation, we used nested repeated 10-fold cross-validation [29,30]. The inner loop of cross-validation was used to determine the best parameters of the classifier (that is, values of parameters yielding the best accuracy for the validation dataset). The outer loop of cross-validation was used for estimating the accuracy of the classifier that was built using the previously found best parameters by testing with an independent set of samples. To account for variance in accuracy estimation, we repeated this entire process (nested 10-fold cross-validation) for 10 different splits of the data into 10 cross-validation testing sets and averaged the results [29].

### Feature/operational taxonomic unit (OTU) selection methods

We used the following feature selection techniques in an effort to improve classification accuracy, alleviate the ‘curse of dimensionality’ and improve interpretability by determining which OTUs were predictive of the different responses:

•Random forest-based backward elimination procedure RFVS [31]: We applied the *varSelRF* implementation of the RFVS method (http://cran.r-project.org/web/packages/varSelRF) with the recommended parameters: *ntree* = 2000, *mtryFactor* = 1, *nodesize* = 1, *fraction.dropped* = 0.2 (a parameter denoting fraction of OTUs with low importance values to be discarded during the backward elimination procedure), and *c.sd* = 0 (a factor that multiplies the standard deviation of error for stopping iterations and choosing the best performing subset of OTUs). We refer to this method as ‘RFVS1.’

•The RFVS procedure as described above, except that *c.sd* = 1 (denoted as ‘RFVS2’): This method differs from RFVS1 in that it performs statistical comparison to return the smallest subset of OTUs with classification accuracy that is not statistically distinguishable from the nominally best one.

•The SVM-based recursive feature elimination method SVM-RFE [32]: To be comparable with the RFVS method, we used the fraction of OTUs that are discarded in the iterative SVM models equal to 0.2. This variable selection method was optimized separately for the employed accuracy metrics. We used implementation of SVM-RFE on top of the libSVM library [25,26].

•A backward elimination procedure based on univariate ranking of OTUs with Kruskal-Wallis one-way non-parametric ANOVA [3] (denoted as ‘KW’): Similarly to SVM-RFE and RFVS, we performed backward elimination by discarding 20% of the OTUs at each iteration. This variable selection method was optimized separately for the employed accuracy metrics. We used implementation of this variable selection procedure on top of the libSVM library [25,26] and Matlab Statistics Toolbox.

We emphasize that all feature selection methods were applied during cross-validation utilizing only the training data and splitting it into smaller training and validation sets, as necessary. This ensures integrity of the model accuracy estimation by protecting against overfitting.

### Accuracy metrics

We used two classification accuracy metrics: the proportion of correct classifications (PCC) and relative classifier information (RCI). The first is easy to interpret and simplifies statistical testing, but is sensitive to the prior class probabilities and does not fully describe the actual difficulty of the classification problem. For example, in the CBH dataset where 357 out of 552 samples are drawn from the skin, a trivial classifier that would always predict the class of maximum prior probability would still obtain PCC = 0.647. On the other hand, a trivial classifier would achieve only PCC = 1/7 = 0.143 in the CS dataset where there are 20 samples from each of the 7 individuals that we want to classify.

The RCI metric is an entropy-based measure that quantifies how much the uncertainty of the decision problem is reduced by the classifier, relative to classifying by simply using the prior probabilities of each class [33]. As such, it corrects for differences in prior probabilities of the diagnostic categories, as well as the number of categories.

The values of both metrics range from 0 to 1, where 0 indicates worst and 1 indicates best classification performance.

### Statistical comparison among classifiers

To test whether the differences in accuracy between the nominally best method (that is, the one with the highest average accuracy) and all remaining algorithms are non-random, we need a statistical comparison of the observed differences in accuracies. We used random permutation testing, as described in [34]. For every algorithm *X*, other than the nominally best algorithm *Y*, we performed the following steps: (I) we defined the null hypothesis (H_0_) to be algorithm *X* is as good as *Y*, that is, the accuracy of the best algorithm *Y* minus the accuracy of algorithm *X* is zero; (II) we obtained the permutation distribution of Δ_*XY*_, the estimator of the true unknown difference between accuracies of the two algorithms under the null hypothesis, by repeatedly swapping the accuracy measures of *X* and *Y* at random for each of the datasets and cross-validation testing sets; (III) we computed the cumulative probability (*P* value) of Δ_*XY*_ being greater than or equal to the observed difference ${\hat{\Delta }}_{\mathrm{XY}}$ over 10,000 permutations. This process was repeated for each of the 10 data splits, and the *P* values were averaged. If the resulting *P* value was smaller than 0.05, we rejected H_0_ and concluded that the data support that algorithm *X* is not as good as *Y* in terms of classification accuracy, and this difference is not due to sampling error. The procedure was run separately for PCC and RCI accuracy metrics.

## Results and Discussion

### Classification without feature/operational taxonomic unit (OTU) selection

**Figure 1:**
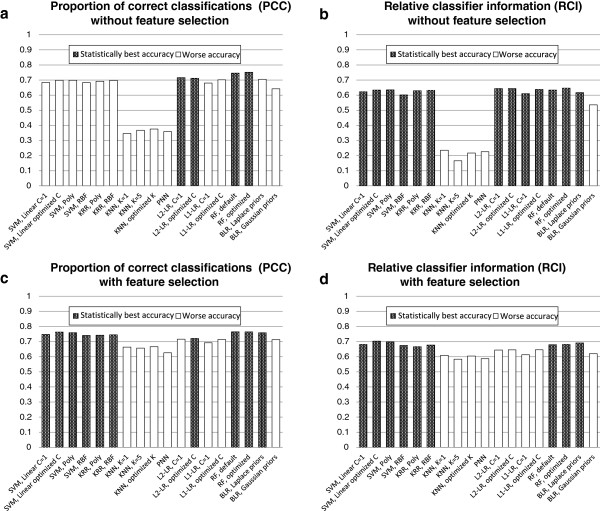
**Accuracies of all classification algorithms averaged over eight datasets.** Panels: (**a**) Proportion of correct classifications (PCC) without feature selection, (**b**) Relative classifier information (RCI) without feature selection, (**c**) PCC with feature selection, and (**d**) RCI with feature selection. The nominally best performing method and methods whose performance cannot be deemed statistically worse than the nominally best performing method are shown as shaded bars; all other methods are shown as empty bars. See text for definition of the PCC and RCI metrics and details of statistical comparison.

Classification accuracy results of experiments without feature/OTU selection, averaged over eight datasets, are provided in Figure 1a,b. Detailed dataset-by-dataset classification accuracy results are shown in Tables 4 and 5. For each classifier, we include the classification performance on each individual dataset, the average performance over all datasets, and the *P* value associated with its statistical comparison against the nominally best performing classifier.

**Table 4:** Classification accuracy without feature/operational taxonomic unit (OTU) selection, measured by proportion of correct classifications (PCC)

**Classifier**	**CBH**	**CS**	**CSS**	**FS**	**FSH**	**BP**	**PDX**	**PBS**	**Averages**	***P *** **values**
*SVM, Linear C = 1*	0.920	0.911	0.583	0.940	0.598	0.354	0.468	0.695	0.684	0.022
*SVM, Linear optimized C*	0.920	0.911	0.622	0.980	0.585	0.383	0.485	0.709	0.699	0.038
*SVM, Poly*	0.920	0.911	0.622	0.980	0.585	0.383	0.484	0.709	0.699	0.036
*SVM, RBF*	0.909	0.904	0.575	0.973	0.575	0.379	0.451	0.700	0.683	0.021
*KRR, Poly*	0.913	0.918	0.581	0.954	0.598	0.377	0.482	0.709	0.692	0.027
*KRR, RBF*	0.923	0.904	0.618	0.967	0.632	0.366	0.467	0.709	0.698	0.030
*KNN, K = 1*	0.496	0.360	0.195	0.451	0.305	0.249	0.419	0.291	0.346	0.002
*KNN, K = 5*	0.713	0.339	0.188	0.397	0.281	0.331	0.393	0.300	0.368	0.001
*KNN, optimized K*	0.714	0.377	0.192	0.325	0.273	0.340	0.409	0.379	0.376	0.001
*PNN*	0.743	0.321	0.216	0.522	0.332	0.325	0.167	0.247	0.359	0.000
*L2-LR, C = 1*	0.934	0.939	0.628	0.982	0.628	0.380	0.515	0.725	0.716	0.084*
*L2-LR, optimized C*	0.933	0.938	0.623	0.978	0.618	0.383	0.502	0.725	0.712	0.067*
*L1-LR, C = 1*	0.929	0.801	0.559	0.975	0.700	0.422	0.384	0.673	0.680	0.018*
*L1-LR, optimized C*	0.928	0.903	0.561	0.981	0.690	0.445	0.412	0.692	0.702	0.039
*RF, default*	0.932	0.955	0.673	0.999	0.744	0.508	0.424	0.730	0.746	0.270*
***RF, optimized***	**0.938**	**0.956**	**0.689**	**0.994**	**0.760**	**0.523**	**0.423**	**0.735**	**0.752**	**-**
*BLR, Laplace priors*	0.927	0.927	0.634	0.962	0.622	0.387	0.452	0.727	0.705	0.042
*BLR, Gaussian priors*	0.921	0.736	0.480	0.966	0.631	0.354	0.410	0.635	0.642	0.008

The nominally best performing classifier on average over all datasets is marked with bold, and *P* values of methods whose performance cannot be deemed statistically worse than the nominally best performing method are marked with “*”. The accuracy of the nominally best performing method for each dataset is underlined.

**Table 5:** Classification accuracy without feature/operational taxonomic unit (OTU) selection, measured by relative classifier information (RCI)

**Classifier**	**CBH**	**CS**	**CSS**	**FS**	**FSH**	**BP**	**PDX**	**PBS**	**Averages**	***P *** **values**
*SVM, Linear C = 1*	0.769	0.918	0.674	0.882	0.749	0.158	0.228	0.602	0.623	0.165*
*SVM, Linear optimized C*	0.771	0.915	0.674	0.958	0.751	0.157	0.241	0.607	0.634	0.294*
*SVM, Poly*	0.771	0.915	0.674	0.958	0.751	0.162	0.241	0.607	0.635	0.299*
*SVM, RBF*	0.689	0.907	0.631	0.942	0.731	0.156	0.202	0.561	0.602	0.059*
*KRR, Poly*	0.765	0.927	0.671	0.911	0.758	0.157	0.230	0.612	0.629	0.206*
*KRR, RBF*	0.774	0.913	0.675	0.935	0.759	0.163	0.242	0.598	0.632	0.265*
*KNN, K = 1*	0.344	0.329	0.377	0.163	0.355	0.167	0.074	0.078	0.236	0.003
*KNN, K = 5*	0.178	0.359	0.277	0.102	0.203	0.056	0.092	0.062	0.166	0.002
*KNN, optimized K*	0.337	0.402	0.354	0.028	0.207	0.089	0.122	0.196	0.217	0.003
*PNN*	0.325	0.292	0.411	0.236	0.342	0.041	0.070	0.089	0.226	0.002
*L2-LR, C = 1*	0.772	0.941	0.670	0.964	0.778	0.161	0.236	0.628	0.644	0.575*
*L2-LR, optimized C*	0.782	0.939	0.680	0.958	0.778	0.163	0.228	0.624	0.644	0.626*
*L1-LR, C = 1*	0.769	0.825	0.635	0.949	0.779	0.163	0.191	0.565	0.610	0.089*
*L1-LR, optimized C*	0.798	0.910	0.664	0.960	0.790	0.174	0.209	0.599	0.638	0.439*
*RF, default*	0.767	0.957	0.671	0.998	0.803	0.173	0.087	0.618	0.634	0.253*
***RF, optimized***	**0.784**	**0.962**	**0.681**	**0.994**	**0.805**	**0.225**	**0.098**	**0.625**	**0.647**	-
*BLR, Laplace priors*	0.759	0.932	0.679	0.922	0.770	0.166	0.090	0.619	0.617	0.085*
*BLR, Gaussian priors*	0.744	0.759	0.496	0.930	0.736	0.077	0.014	0.529	0.536	0.008

The nominally best performing classifier on average over all datasets is marked with bold, and *P* values of methods whose performance cannot be deemed statistically worse than the nominally best performing method are marked with “*”. The accuracy of the nominally best performing method for each dataset is underlined.

Notably, we obtain different results depending on which performance metric we use. In both cases, random forests with optimized parameters is the nominally best performing classifier. When using PCC as a performance metric, the nominally best performing classifier is statistically significantly better than all other classifiers except for L_2_-regularized logistic regression and random forests with default parameters. However, when using RCI as a performance metric, the only classifiers which are statistically significantly *worse* than the nominally best performing classifier are the KNN-based methods, PNN and BLR with Gaussian priors. Therefore, several classifiers which are significantly worse when using the somewhat naive PCC metric are comparable when using the more descriptive RCI metric (Figure 1a,b).

### Classification with feature/operational taxonomic unit (OTU) selection

Classification accuracy results of experiments with feature/OTU selection, averaged over 8 datasets, are provided in Figure 1c,d. Detailed dataset-by-dataset classification accuracy results are shown in Tables 6 and 7. The tables present results for the best performing feature selection method for each classifier/dataset combination under the operating assumption that practitioners will optimize the choice of feature selection method for each dataset separately (using cross-validation or other suitable protocols). As before, for each classifier and feature selection method we include the performance on each individual dataset, the average performance over all datasets, and the *P* value associated with the statistical comparison test against the nominally best performing classifier.

**Table 6:** Classification accuracy with feature/operational taxonomic unit (OTU) selection, measured by proportion of correct classifications (PCC)

**Classifier**	**Best FS Method**	**CBH**	**CS**	**CSS**	**FS**	**FSH**	**BP**	**PDX**	**PBS**	**Averages**	***P *** **values**
*SVM, Linear C = 1*	*SVM-RFE*	0.900	0.941	0.610	0.965	0.719	0.524	0.558	0.759	0.747	0.319*
*SVM, Linear optimized C*	*SVM-RFE*	0.952	0.935	0.631	0.985	0.754	0.534	0.553	0.761	0.763	0.535*
*SVM, Poly*	*SVM-RFE*	0.950	0.929	0.633	0.987	0.742	0.528	0.551	0.754	0.759	0.460*
*SVM, RBF*	*SVM-RFE*	0.941	0.918	0.617	0.987	0.693	0.518	0.523	0.727	0.741	0.179*
*KRR, Poly*	*KW*	0.909	0.933	0.623	0.949	0.749	0.547	0.514	0.713	0.742	0.199*
*KRR, RBF*	*KW*	0.929	0.939	0.634	0.970	0.737	0.537	0.504	0.714	0.745	0.248*
*KNN, K = 1*	*RFVS2*	0.930	0.760	0.563	0.971	0.623	0.421	0.443	0.596	0.663	0.011
*KNN, K = 5*	*RFVS2*	0.930	0.724	0.529	0.943	0.656	0.434	0.434	0.609	0.657	0.009
*KNN, optimized K*	*RFVS2*	0.935	0.754	0.552	0.963	0.648	0.422	0.432	0.620	0.666	0.011
*PNN*	*RFVS2*	0.906	0.781	0.560	0.956	0.623	0.130	0.449	0.604	0.626	0.006
*L2-LR, C = 1*	*ALL*	0.934	0.939	0.628	0.982	0.628	0.380	0.515	0.725	0.716	0.047
*L2-LR, optimized C*	*KW*	0.921	0.948	0.650	0.836	0.739	0.499	0.464	0.711	0.721	0.089*
*L1-LR, C = 1*	*RFVS1*	0.922	0.818	0.589	0.968	0.706	0.449	0.395	0.687	0.692	0.020
*L1-LR, optimized C*	*RFVS1*	0.934	0.909	0.611	0.993	0.710	0.442	0.418	0.697	0.714	0.048
***RF, default***	***RFVS1***	**0.954**	**0.950**	**0.704**	**0.991**	**0.745**	**0.550**	**0.479**	**0.746**	**0.765**	-
*RF, optimized*	*RFVS1*	0.954	0.950	0.695	0.996	0.746	0.548	0.479	0.741	0.764	0.498*
*BLR, Laplace priors*	*SVM-RFE*	0.946	0.929	0.639	0.991	0.759	0.521	0.537	0.739	0.758	0.465*
*BLR, Gaussian priors*	*KW*	0.926	0.856	0.557	0.980	0.728	0.525	0.426	0.701	0.713	0.043

The nominally best performing classifier on average over all datasets is marked with bold, and *P* values of methods whose performance cannot be deemed statistically worse than the nominally best performing method are marked with “*”. The accuracy of the nominally best performing method for each dataset is underlined.

**Table 7:** Classification accuracy with feature/ operational taxonomic unit (OTU) selection, measured by relative classifier information (RCI)

**Classifier**	**Best FS Method**	**CBH**	**CS**	**CSS**	**FS**	**FSH**	**BP**	**PDX**	**PBS**	**Averages**	***P *** **values**
*SVM, Linear C = 1*	*SVM-RFE*	0.719	0.952	0.691	0.929	0.813	0.334	0.337	0.674	0.681	0.191*
***SVM, Linear optimized C***	***SVM-RFE***	**0.852**	**0.946**	**0.723**	**0.971**	**0.840**	**0.314**	**0.325**	**0.653**	**0.703**	-
*SVM, Poly*	*SVM-RFE*	0.845	0.941	0.716	0.969	0.840	0.316	0.323	0.644	0.699	0.369*
*SVM, RBF*	*SVM-RFE*	0.813	0.925	0.683	0.972	0.813	0.286	0.290	0.611	0.674	0.089*
*KRR, Poly*	*SVM-RFE*	0.759	0.939	0.683	0.931	0.800	0.297	0.290	0.626	0.666	0.061*
*KRR, RBF*	*SVM-RFE*	0.807	0.935	0.687	0.944	0.801	0.297	0.316	0.633	0.677	0.097*
*KNN, K = 1*	*RFVS2*	0.830	0.779	0.657	0.939	0.736	0.168	0.251	0.510	0.609	0.015
*KNN, K = 5*	*RFVS2*	0.774	0.744	0.625	0.884	0.736	0.153	0.224	0.522	0.583	0.008
*KNN, optimized K*	*RFVS2*	0.829	0.773	0.652	0.914	0.736	0.179	0.221	0.531	0.604	0.014
*PNN*	*RFVS2*	0.726	0.798	0.629	0.907	0.730	0.167	0.227	0.516	0.587	0.012
*L2-LR, C = 1*	*ALL*	0.772	0.941	0.670	0.964	0.778	0.161	0.236	0.628	0.644	0.027
*L2-LR, optimized C*	*SVM-RFE*	0.780	0.940	0.692	0.837	0.811	0.234	0.257	0.612	0.645	0.034
*L1-LR, C = 1*	*RFVS1*	0.742	0.836	0.642	0.934	0.771	0.183	0.213	0.584	0.613	0.011
*L1-LR, optimized C*	*RFVS1*	0.786	0.914	0.696	0.985	0.784	0.166	0.238	0.598	0.646	0.033
*RF, default*	*RFVS1*	0.840	0.952	0.712	0.982	0.819	0.266	0.213	0.648	0.679	0.179*
*RF, optimized*	*RFVS1*	0.842	0.956	0.714	0.994	0.810	0.264	0.216	0.649	0.681	0.196*
*BLR, Laplace priors*	*SVM-RFE*	0.822	0.932	0.692	0.982	0.824	0.317	0.318	0.640	0.691	0.313*
*BLR, Gaussian priors*	*RFVS2*	0.761	0.855	0.625	0.968	0.770	0.208	0.202	0.570	0.620	0.018

The nominally best performing classifier on average over all datasets is marked with bold, and *P* values of methods whose performance cannot be deemed statistically worse than the nominally best performing method are marked with “*”. The accuracy of the nominally best performing method for each dataset is underlined.

For many methods, there is a significant improvement using feature/OTU selection prior to performing classification. For both accuracy metrics, there is no statistically significant difference between the performance of SVMs, kernel ridge regression and random forests. The improvement in performance due to feature selection is especially pronounced in the case of KNN and PNN, which is consistent with the general understanding that these methods are sensitive to a large number of irrelevant features. However, KNN and PNN are still among the worst performing classifiers (Figure 1c,d).

The number of OTUs selected on average across the 10 data splits and 10 cross-validation training sets is provided in Table 8. We note that feature/OTU selection was optimized for each accuracy metric separately to the extent that the feature selection methods allowed it. Specifically, we used the same accuracy metric for evaluating model accuracy internally in the SVM-RFE and KW feature selection methods as for evaluating the final classification accuracy on the testing sets. In the case of RFVS, we used the package provided by the authors which only allows the use of PCC for evaluation of model accuracy for the different subsets of variables [31]; hence, the same sets of features were used in both the PCC and RCI benchmarks. Finally, we note that in the present work we focus exclusively on classification accuracy and do not incorporate the number of selected OTUs in the comparison metrics because there is no well-defined trade-off between the number of selected OTUs and the classification accuracy in the datasets studied.

**Table 8:** Number of features/operational taxonomic units (OTUs) selected on average across ten data splits and ten cross-validation training sets

**Dataset**	**No OTU selection**	**Optimized for PCC**				**Optimized for RCI**	
		**SVM-RFE**	**KW**	**RFVS1**	**RFVS2**	**SVM-RFE**	**KW**
*CBH*	6979	259	1191	20	50	285	1359
*CS*	2543	469	370	215	805	474	370
*CSS*	4793	896	935	51	211	1166	1262
*FS*	1217	9	8	8	8	9	8
*FSH*	1217	101	128	38	108	126	142
*BP*	13503	453	416	37	204	1223	1276
*PDX*	74018	5127	8308	136	492	8164	7400
*PBS*	74018	2633	4347	89	687	4568	12188

## Conclusions

In this work, we conducted a thorough evaluation to identify the most accurate machine learning algorithms for multicategory classification from microbiomic data. We evaluated 18 algorithms for multicategory classification, 5 feature selection methods and 2 accuracy metrics on 8 different classification tasks with human microbiomic data. We found that for the most part, SVMs, random forests, kernel ridge regression, and Bayesian logistic regression with Laplace priors provided statistically similar levels of classification accuracy. On the other hand, we also found that K-nearest neighbors and probabilistic neural networks were significantly outperformed by the other techniques.

The results of this work also highlight the large variation in difficulty across the different classification tasks. Tasks that involve classifying body sites, body habitats or subjects yield much higher accuracy rates than those which involve predicting the correct diagnosis, which are arguably more useful for real-life clinical applications. However, considering that the use of microbiomics for disease diagnosis has so far been relatively unexplored, the fact that we can still produce predictions that are better than random is encouraging.

The present results are relevant to the extent that the datasets employed are representative of the characteristics of microbiomic datasets in common use. We believe that we provided a dataset catalogue with broadly relevant characteristics. Of course, analysts when using the present benchmark comparison results to inform their analyses, should consider the degree of similarity of their datasets to the datasets in the study.

Finally, we mention that the results of this work may not be limited to microbiomic applications, and they might also apply to other similar classification tasks with next-generation DNA sequencing data. For example, classification with metagenomic surveys, in which the input features correspond to abundances of genes or gene families from different organisms, would be an interesting direction for future work.

## Abbreviations

BLR: Bayesian logistic regression (machine learning method); KNN: K-nearest neighbors (machine learning method); KRR: kernel ridge regression (machine learning method); L1-LR: regularized logistic regression by an L_1_ penalty (machine learning method); L2-LR: regularized logistic regression by an L_2_ penalty (machine learning method); OTU: operational taxonomic unit; PCC: proportion of correct classifications (classification accuracy metric); PNN: probabilistic neural networks (machine learning method); QIIME: Quantitative Insights Into Microbial Ecology; RCI: relative classifier information (classification accuracy metric); RF: random forests (machine learning method); SVM: support vector machine (machine learning method)

## Competing interests

The authors declare that they have no competing interests.

## Authors’ contributions

AS and AVA conceived the research study and designed the methods and experiments. MH, VN, KK, ZL, LY prepared the data, implemented the methods and conducted all experiments and data analysis. AS, AVA, MH, KK, VN participated in the interpretation of the results. All authors have contributed to, read, and approved the final manuscript.

## Supplementary Material

Additional file 1 Operational taxonomic unit table and sample labels for the CBH dataset. Click here for file

Additional file 2 Operational taxonomic unit table and sample labels for the CS dataset. Click here for file

Additional file 3 Operational taxonomic unit table and sample labels for the CSS dataset. Click here for file

Additional file 4 Operational taxonomic unit table and sample labels for the FS dataset. Click here for file

Additional file 5 Operational taxonomic unit table and sample labels for the FSH dataset. Click here for file

Additional file 6 Operational taxonomic unit table and sample labels for the BP dataset. Click here for file

Additional file 7 Operational taxonomic unit table and sample labels for the PBS dataset. Click here for file

Additional file 8 Operational taxonomic unit table and sample labels for the PDX dataset. Click here for file
